# Prevalence, Awareness, and Associated Factors of Airflow Obstruction in Russia: The Ural Eye and Medical Study

**DOI:** 10.3389/fpubh.2019.00350

**Published:** 2019-11-20

**Authors:** Mukharram M. Bikbov, Gyulli M. Kazakbaeva, Rinat M. Zainullin, Venera F. Salavatova, Inga I. Arslangareeva, Songhomitra Panda-Jonas, Timur R. Gilmanshin, Nikolai A. Nikitin, Svetlana R. Mukhamadieva, Dilya F. Yakupova, Renat I. Khikmatullin, Said K. Aminev, Ildar F. Nuriev, Artur F. Zaynetdinov, Yulia V. Uzianbaeva, Jost B. Jonas

**Affiliations:** ^1^Ufa Eye Research Institute, Ufa, Russia; ^2^Department of Ophthalmology, Medical Faculty Mannheim of the Ruprecht-Karls-University of Heidelberg, Heidelberg, Germany

**Keywords:** airflow obstruction, chronic obstructive pulmonary disease, asthma, forced expiratory volume, household air pollution, occupational particulates, population-based study, ural eye and medical study

## Abstract

**Background:** Although chronic obstructive pulmonary disease and asthma belong to the most important causes of disability and death in all world regions, data about the prevalence of airflow obstruction and asthma in Russia and the associated parameters have been scarce so far. We therefore assessed the prevalence of airflow obstruction and asthma in a Russian population.

**Methods:** The population-based Ural Eye and Medical Study, conducted in a rural and urban region of Bashkortostan/Russia, included 5,392 participants (mean age: 58.6 ± 10.6 years; range: 40–94 years) out of 7,328 eligible individuals. Airflow obstruction was defined spirometrically and asthma by self-reported diagnosis.

**Results:** Airflow obstruction was present in 369 individuals (6.8%; 95% confidence interval (CI): 6.2, 7.5) with an awareness rate of 63.4% (95%CI: 58.5, 68.4) and known duration of 19.5 ± 15.8 years (median: 16 years). Prevalence of undiagnosed airflow obstruction was 2.6% (95%CI: 2.2, 3.1). Higher prevalence of airflow obstruction was associated (multivariable analysis) with higher prevalence of current smoking [*P* < 0.001; odds ratio (OR): 2.91; 95%CI: 1.76, 4.83] and number of cigarette package years (*P* < 0.001; OR: 1.03; 95%CI: 1.02, 1.08), female gender (*P* = 0.03; OR: 1.42; 95%CI: 1.04, 1.93), urban region (*P* = 0.003; OR: 1.43; 95% CI: 1.12, 1.79), higher prevalence of cardiovascular diseases/stroke (*P* < 0.001; OR: 1.86; 95%CI: 1.45, 2.39), higher depression score (*P* = 0.002; OR: 1.05; 95%CI: 1.02, 1.08), and lower physical activity (*P* = 0.01; OR: 0.71; 95%CI: 0.54, 0.93). Asthma prevalence (2.6%; 95%CI: 2.0, 3.1; known duration: 17.2 ± 15.0 years) was associated with less alcohol consumption (OR: 0.53; 95%CI: 0.33, 0.87; *P* = 0.01), higher depression score (OR: 1.08; 95%CI: 1.03, 1.12; *P* < 0.001), and urban region (OR: 0.68; 95CI: 0.49, 0.95; *P* = 0.0.03).

**Conclusions:** In this Russian population aged 40+ years, the prevalence of airflow obstruction was 6.8% with an awareness rate of 63.4% and smoking as main risk factor. Asthma prevalence was 2.6%.

## Introduction

Chronic obstructive pulmonary disease (COPD) and asthma belong to the most frequent reasons of death and disability in all world regions. The Global Burden of Disease Study 2017 ranked COPD in the list of the most frequent causes of global DALYs (disability-adjusted life-years) for both sexes at position #6 in 2017, at position #8 in 2007, and at position #7 in 1990 (1). In a parallel manner, COPD ranked at position #11 in 1990 and in 2007 and at position #7 in 2017 in the list of the leading causes of years of life lost (YLLs) globally (2). According to the Global Burden of Diseases Study 2017, 299 million individuals were affected by COPD in 2017, and 3.2 million people died in 2017 from COPD worldwide (2, 3).

In a similar manner, 0.40 million people died from asthma in 2015 worldwide, and the prevalence of asthma increased globally by 12.6%, whereas the age-standardized prevalence decreased by 17.7% (4). The main predisposing factors for COPD were smoking and ambient particulate matter while household air pollution, occupational particulates, ozone and secondhand smoke also played a role. Together, these risks explained 73.3% of DALYs due to COPD. In the same survey, smoking and occupational asthmagens as the only quantified risk factors for asthma accounted for 16.5% of DALYs due to asthma (4).

Despite the high importance of COPD and asthma for public health, data about the prevalence of COPD or airflow obstruction and asthma in Russia and parameters associated with the occurrence of both diseases in Russia has remained scarce so far (5–7). We therefore explored the prevalence of airflow obstruction and asthma in a population in Russia and assessed associations between these diseases and other major risk factors. With the population of Russia including many ethnicities, we chose the Russian republic of Bashkortostan as study site, since the population of Bashkortostan includes Russians and other ethnic groups with different cultural backgrounds (8, 9).

## Methods

We conducted the Ural Eye and Medical Study in the city of Ufa as the capital of the republic of Bashkortostan and in a rural region in a distance of 65 km to Ufa. Ufa is located about 1,400 km East of Moscow at the Southwestern end of the Ural Mountains. An age of 40 years or older and living in the study regions were the only inclusion criteria of the study. The Ethics Committee of the Academic Council of the Ufa Eye Research Institute approved the study protocol and all participants gave an informed written consent.

Trained social workers conducted an interview which included more than 250 standardized questions on the socioeconomic background, smoking habits and alcohol consumption, physical activity, depression and anxiety, and known diagnosis and therapy of major diseases (Supplemental Table 1). The questionnaire in particular included questions on chronic cough, breathlessness on exertion, sputum production, frequent exacerbations of bronchitis, and a history of exposure to risk factors, especially tobacco smoke, occupational dusts, home cooking and biomass fuels (Table 1) (10). The study methods further included anthropometry, blood pressure measurement, handgrip dynamometry, spirometry, and biochemical analysis of blood samples taken under fasting conditions. We assessed the presence of depression by applying the Center for Epidemiologic Studies Depression Scale (CES-D) Scoresheet, and we explored trait and state anxiety by using the State-Trait Anxiety Inventory (STAI). We defined the levels of blood pressure using the guidelines of the American Heart Association, and diabetes mellitus by a fasting serum glucose concentration of ≥7.0 mmol/L or a self-reported history of physician-based diagnosis or therapy of diabetes mellitus (11). We applied the Guidelines for Accurate and Transparent Health Estimates Reporting (GATHER statement guidelines) (12). We have described the study design in detail recently (8, 9, 13, 14).

**Table 1:** Spirometric measurements and clinical signs of airflow obstruction (mean ± standard deviations; frequency and 95% confidence intervals) in the Ural Eye ad Medical Study stratified by the presence of airflow obstruction (defined by a ratio of forced expiratory volume in 1 s divided by the mean forced vital capacity of <0.7).

	**Reference category or unit of measure**	**Forced expiratory volume in 1 s (FEV1)/mean forced vital capacity (FVC) <0.7**	**FEV1/FVC ≥0.7**	*P*-Value^*^
*n*		369	5,023	
Forced vital capacity (FVC)	L	1.86 ± 0.75	2.25 ± 0.51	<0.001
Forced expiratory volume in 1 s (FEV1)	L	1.23 ± 0.51	1.95 ± 0.44	<0.001
FEV1/FVC	Ratio	0.66 ± 0.02	0.87 ± 0.04	<0.001
Tidal volume	L	0.41 ± 0.16	0.54 ± 0.14	<0.001
Do you have chronic obstructive pulmonary disease?	Yes/No	234%/135%	101%/4922%	<0.001
Since when do you have airflow obstruction?	Years	19.5 ± 15.8	15.6 ± 13.2	0.03
Do you have asthma?	Yes/No	41/328	101/4,922	<0.001
Since when do you have asthma?	Years	18.1 ± 14.2	16.8 ± 15.3	0.63
*n*		163	3,035	
How many times a day do you cough	Categories of: Never/1–3 times/4−6 times/>6 times	43%/38%/10%/9%	70%/25%/3%/2%	<0.001
How often have you have sputum during cough?	Categories of: Never/1–3 times/4−6 times/>6 times	58%/28%/6%/8%	82%/16%/1%/1%	<0.001
How often have you been without sputum cough?	Categories of: Never/1–3 times/4−6 times/>6 times	77%/17%/3%/3%	88%/11%/1%/0%	0.001
How often you do catch a cold in the winter?	Categories of: Never/1-3 times/4−6 times/>6 times	25%/68%/7%/1%	43%/55%/2%/0%	<0.001
Is difficult for you to breathe when climbing stairs?	No/Yes	29%/71%	53%/47%	<0.001
After how many steps do you have difficulty in breathing?	20–30/31–40/>40	59%/32%/9%	32%/43%/25%	0.001
Do you have a fireplace or stove with an open fire at home?	No/Yes	96%/4%	96%/4%	0.68
Is there smoke at your workplace?	No/Yes	94%/6%	96%/4%	0.22
Is there dust at your workplace?	No/Yes	90%/10%	94%/6%	0.03

* *Student-t-test, Chi-square test or analysis of variance ANOVA*.

All participants underwent a pulmonary function test by spirometric measurement (spirometry device: Riester spirotest, Riester Company, Jungingen, Germany) of the forced expiratory volume in 1 s (FEV1) and of the forced vital capacity (FVC). The FEV1 was defined as the greatest volume of air that could be breathed out in the first second after in-breathing as deep as possible, and FVC was defined as the greatest volume of air that could be breathed out in a single breath as hard as possible, for as long as possible, preferably for at least 6 s. We additionally measured the tidal volume (Table 1) (14). We applied the recommendations made by the American Thoracic Society and European Respiratory Society Task Force (15–17), except for not using a bronchodilator. Since we did not apply a bronchodilator, the measurements were pre-bronchodilator readings. In agreement with the Global Initiative of Chronic Obstructive Lung Disease, airflow obstruction was defined applying a spirometry-based definition with a cut-off value of the FEV1/FVC ratio of <0.7 (10). Using the recommendation made by Hankinson et al. we additionally calculated the lower limit of normal (LLN) for the spirometric measurements to define the presence of airflow obstructions (18). Asthma was defined by a self-reported diagnosis of physician-made diagnosis of asthma.

Using a statistical software program [SPSS (Statistical Package for Social Science) version 25.0; IBM-SPSS Inc., Chicago, USA], we first calculated the prevalence of airflow obstruction and showed the results as mean and 95% confidence intervals (CI). We then assessed differences between the group of participants with airflow obstruction and the individuals without airflow obstruction. We finally conducted a multivariable binary regression analysis with the prevalence of airflow obstruction as dependent variable and as independent variables all those parameters, which differed significantly between the group with vs. without airflow obstruction. We determined the odds ratios (OR) and their 95% confidence intervals (CI). All *P*-values were two-sided and we considered them statistically significant if their values were <0.05.

## Results

Out of 7,328 eligible individuals, the Ural Eye and Medical Study included 5,899 (80.5%) study participants. Ranging between 40 and 94 years, the mean age was 59.0 ± 10.7 years. The demographic data of the study population did not vary significantly from the data of the Russian census carried out in 2010 (http://www.gks.ru/). The present investigation consisted of 5,392 (91.4%) individuals [2,449 (45.4%) men] for whom spirometric measurements were available. The group of study participants with spirometric measurements as compared with the group of individuals without spirometric measurements was significantly younger (58.6 ± 10.6 years vs. 63.1 ± 11.3 years; *P* < 0.001), and had a significantly higher proportion of men [2,449 (45.4%) men/2,943 (54.6%) women vs. 131 (25.8%) men/376 (74.2%) women; *P* < 0.001]. We assessed the symptoms of airflow obstruction, biomass use and workplace exposures for only 3,198 participants, while we performed spirometry for all 5,391 individuals (Table 1). The reason was that the questions on obstructive airflow symptoms were included into the questionnaire after the study had started.

**Figure 1:**
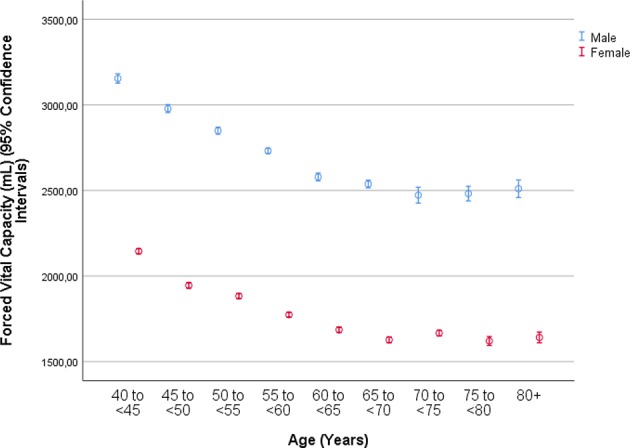
Graph showing the distribution of the forced vital capacity, stratified by age and gender, in the Ural Eye and Medical Study.

**Figure 2:**
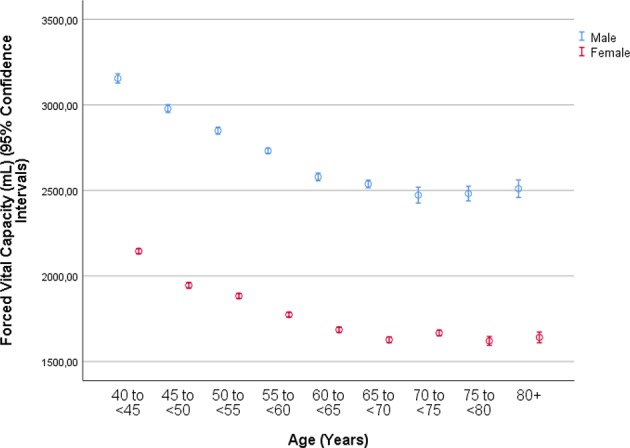
Graph showing the distribution of the forced expiratory volume in 1 s, stratified by age and gender, in the Ural Eye and Medical Study.

**Figure 3:**
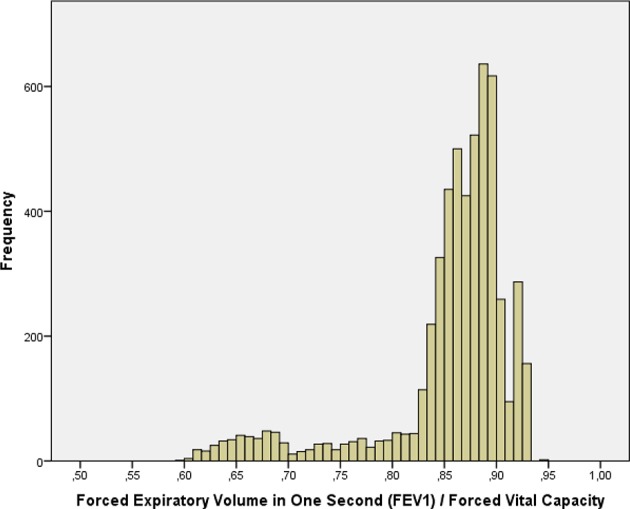
Histogram showing the distribution of the ratio of the forced expiratory volume in 1 s divided by the forced vital capacity in the Ural Eye and Medical Study.

The mean forced vital capacity (FVC) was 2.22 ± 0.54 L, the mean vital capacity measured 2.53 ± 0.60 L, the forced expiratory volume in 1 s (FEV1) was 1.90 ± 0.48 L, and the ratio of FEV1 to FVC averaged 0.86 ± 0.07 L/s (median: 0.87; range: 0.59, 0.94) (Figures 1–3, Table 2).

**Table 2:** Spirometric measurements in the Ural Eye and Medical Study, stratified by sex, and age.

**Age group (Years)**	***n***	**Forced expiratory volume in 1 s (FEV1)/forced vital capacity (FVC) (mean ± standard deviation)**	**Forced expiratory volume in 1 s (FEV1) (mL) (mean ± standard deviation)**	**Forced vital capacity (FVC) (mL) (mean ± standard deviation)**
**MEN**				
40–44	209	0.82 ± 0.08	2,603 ± 340	3,155 ± 199
45–49	350	0.84 ±0.07	2,503 ± 301	2,978 ± 221
50–54	422	0.84 ± 0.07	2,404 ± 284	2,849 ± 211
55–59	471	0.85 ± 0.06	2,318 ± 243	2,731 ± 179
60–64	382	0.85 ± 0.07	2,193 ± 277	2,579 ± 222
65–69	273	0.86 ± 0.06	2,179 ± 247	2,538 ± 188
70–74	124	0.85 ± 0.07	2,106 ± 303	2,473 ± 261
75–79	150	0.86 ±0.07	2,144 ± 325	2,482 ± 267
80+	68	0.86 ± 0.05	2,173 ± 242	2,510 ± 213
**WOMEN**				
40–44	252	0.87 ± 0.04	1,875 ± 137	2,145 ± 122
45–49	361	0.87 ± 0.06	1,690 ± 194	1,945 ± 153
50–54	465	0.86 ± 0.05	1,630 ± 188	1,883 ± 169
55–59	499	0.86 ± 0.06	1,534 ± 204	1,773 ± 165
60–64	448	0.86 ± 0.07	1,453 ± 205	1,686 ± 164
65–69	429	0.86 ± 0.07	1,407 ± 234	1,627 ± 180
70–74	182	0.87 ± 0.05	1,457 ± 152	1,667 ± 118
75–79	202	0.86 ± 0.08	1,404 ± 247	1,620 ± 188
80+	105	0.87 ± 0.07	1,434 ± 205	1,641 ± 162

Airflow obstruction defined by a FEV1/FVC ratio <0.7 was present in 369 individuals indicating a prevalence of 6.8% (95%CI: 6.2, 7.5) (Supplemental Table 1, Table 3). The awareness rate of having airflow obstruction was 234/369 or 63.4% (95%CI: 58.5, 68.4). For 213 individuals who could remember when their airflow obstruction was detected, the known duration of airflow obstruction was 19.5 ± 15.8 years (median: 16 years; range: 0–69 years). For 156 individuals, the known duration of airflow obstruction could not be well-remembered. The prevalence of undiagnosed airflow obstruction was 135/5,158 or 2.6% (95%CI: 2.2, 3.1): 4.8% (95%CI: 3.9, 5.7) in men and 0.8% (95%CI: 0.4, 1.1) in women. All of the 369 individuals with airflow obstruction had a FEV1/FVC ratio larger than 0.50.

**Table 3:** Prevalence of airflow obstruction disease and asthma in the Ural Eye and Medical Study, stratified by sex and age.

**Age group (Years)**	***n***	**Airflow obstruction prevalence (%)**	**95% confidence intervals**	**Asthma (%)**	**95% confidence intervals**
**MEN**					
40–44	209	17.2	12.1, 22.4	1.4	0.0, 3.0
45–49	350	9.14	6.11, 12.2	0.9	0.0, 2.0
50–54	422	6.87	4.45, 9.30	2.8	1.0, 4.0
55–59	471	5.10	3.10, 7.09	1.3	0.0, 2.0
60–64	382	6.55	4.05, 9.04	2.4	1.0, 4.0
65–69	273	6.23	3.34, 9.11	0.7	0.0, 2.0
70–74	124	7.26	2.63, 11.9	6.5	2.0, 11.0
75–79	150	8.00	3.61, 12.4	4.7	1.0, 8.0
80+	68	2.94	−1.18, 7.06	1.5	−1.0, 4.0
**WOMEN**					
40–44	252	1.59	0.03, 3.14	0.4	0.0, 1.0
45–49	361	4.99	2.73, 7.24	2.5	1.0, 4.0
50–54	465	3.87	2.11, 5.63	1.9	1.0, 3.0
55–59	499	6.41	4.26, 8.57	2.6	1.0, 4.0
60–64	448	7.37	4.94, 9.79	3.3	2.0, 5.0
65–69	429	9.56	6.76, 12.4	4.4	2.0, 6.0
70–74	182	3.30	0.7, 5.92	3.8	1.0, 7.0
75–79	202	11.9	7.38, 16.4	8.4	5.0, 12.0
80+	105	6.67	1.82, 11.5	1.0	−1.0, 3.0

Airflow obstruction defined by a FEV1/FVC ratio smaller than the lower limit of normal was present in 315 individuals with a prevalence of 5.8% (95%CI: 5.2, 6.5). The awareness rate of having airflow obstruction was 199/315 or 63.2% (95%CI: 57.8, 68.5). Using the definition of the LLN method, the prevalence of undiagnosed airflow obstruction in the study population was 116/5,392 or 2.2% (95%CI: 1.8, 2.5): 3.6% (95%CI: 2.9, 4.3) in men and 1.0% (95%CI: 0.6, 1.3) in women.

Within the ethnic group of Russians (*n* = 1,185; 508 men, 677 women) with a mean age of 60.1 ± 11.1 years, airflow obstruction was present in 101 individuals [8.5% (95%CI: 6.9, 10.1) with an awareness rate of 65/101 or 64.4% (95%CI: 54.9, 73.9)] and a known duration of airflow obstruction of 18.2 ± 14.5 years (median: 16 years; range: 1–69 years). Within the Russian subgroup, the prevalence of undiagnosed airflow obstruction was 36/1,120 (3.2%; 95%CI: 2.2, 4.3): 5.1% (95%CI: 3.1, 7.0) in men and 1.8% (95%CI: 0.7, 2.8) in women. Comparing the Russian group with the non-Russian group revealed that the airflow obstruction prevalence was significantly (*P* = 0.01) higher in the Russian group [8.5% (95%CI: 6.9, 10.1) vs. 6.4% (95%CI: 5.6, 7.1)], while the awareness rate (*P* = 0.90) did not differ markedly between the ethnic groups [64.4% (95%CI: 54.9, 73.9) vs. 63.1% (95%CI: 57.2, 68.9)].

In univariate analysis, the airflow obstruction group differed from the group without airflow obstruction in the demographic parameters such as gender and region of habitation, anthropometric factors such as body mass index, socioeconomic parameters such as level of education, results of the biochemical blood analysis, and parameters related to physical activity, medical history, diet, smoking, and subjective hearing loss (Supplemental Table 1). In addition, the airflow obstruction group as compared to the group without airflow obstruction showed a higher prevalence of coughing (*P* < 0.001) and having sputum during coughing (*P* < 0.001), higher prevalence of catching a cold in the winter (*P* < 0.001), dyspnea (*P* < 0.001), and higher occurrence rate of dust at the working place (Supplemental Table 1).

The multivariable regression analysis included the occurrence of airflow obstruction as dependent variable and as independent variables all those parameters that were significantly associated with the prevalence of airflow obstruction in the univariate analysis. After dropping in a step-by-step manner those independent parameters which had lost the statistical significance of their association with the airflow obstruction prevalence, a higher airflow obstruction prevalence was associated with a higher prevalence of current smoking and higher number of cigarette package years, female gender, urban region of habitation, higher depression score, higher prevalence of history of arthritis, and cardiovascular diseases including stroke, and higher prevalence of ownership of a telephone, lower prevalence of vigorous activities during leisure time, and lower serum concentration of creatinine (Table 4). If the parameter of cigarette package years was dropped, the odds ratio for current smoking increased to 9.12 (95%CI: 6.77, 12.3). If age or body height were added to model, each parameter was not significantly (*P* = 0.73 and *P* = 0.56, respectively) associated with the prevalence of airflow obstruction.

**Table 4:** Associations (multivariate analysis) of the prevalence of airflow obstruction in both sexes in the Ural Eye and Medical Study, with the prevalence of airflow obstruction as the dependent variable and all other listed parameters as independent variable.

**Parameter**	**Reference category or unit of measure**	***P*-value**	**Odds ratio**	**95% confidence interval**
Current smoking	No/Yes	<0.001	2.91	1.76, 4.83
Cigarette package years	Number	<0.001	1.03	1.02, 1.04
Men/Women	Men, 1; Women, 2	0.03	1.42	1.04, 1.93
Rural/urban region of habitation	Rural, 1; Urban, 2	0.003	1.43	1.12,1.79
Depression score	Number	0.002	1.05	1.02, 1.08
History of arthritis	No/Yes	<0.001	1.74	1.35, 2.24
History of cardiovascular diseases including stroke	No/Yes	<0.001	1.86	1.45, 2.39
Serum concentration of creatinine	mmol/L	0.002	0.99	0.99, 0.99
Ownership of telephone	No/Yes	0.002	2.02	1.31, 3.12
Vigorous activity during leisure time	No/Yes	0.01	0.71	0.54, 0.93

If the LLN-based diagnosis of airflow obstruction was used a higher prevalence of airflow obstruction was associated with a higher prevalence of current smoking (OR: 4.66; 95%CI: 3.01, 7.20; *P* < 0.001) and higher number of cigarette package years (OR: 1.02; 95%CI: 1.01, 1.03; *P* < 0.001), higher depression score (OR: 1.06; 95%CI: 1.03, 1.09; *P* < 0.001), higher prevalence of history of arthritis (OR: 1.58; 95%CI: 1.25, 1.98; *P* < 0.001), and cardiovascular diseases including stroke (OR: 1.86; 95%CI: 1.48, 2.33; *P* < 0.001), and higher prevalence of ownership of a telephone (OR: 1.56; 95%CI: 1.10, 2.21; *P* = 0.01), lower prevalence of vigorous activities during leisure time (OR: 0.76; 95%CI: 0.60, 0.97; *P* = 0.03), and lower serum concentration of creatinine (OR: 0.99; 95%CI: 0.98, 0.99; *P* < 0.001). If age or body height were added to model, each parameter was not significantly (*P* = 0.16 and *P* = 0.88, respectively) associated with the prevalence of airflow obstruction.

If the parameter of a history of menopause was added to the model in women, telephone ownership (*P* = 0.22), cigarette package years (*P* = 0.51), and vigorous activity in leisure time (*P* = 0.054) were no longer significantly associated with airflow obstruction prevalence, so that in the final model, a higher prevalence of airflow obstruction in women was associated with higher prevalence of current smoking (*P* < 0.001), urban region of habitation, higher prevalence of history of menopause, arthritis and cardiovascular diseases including stroke, higher depression score, lower serum concentration of creatinine (Table 5). If age or body height were added to model, each parameter was not significantly (*P* = 0.38 and *P* = 0.85, respectively) associated with the prevalence of airflow obstruction.

**Table 5:** Associations (multivariate analysis) of the prevalence of airflow obstruction in women in the Ural Eye and Medical Study, with the prevalence of airflow obstruction as the dependent variable and all other listed parameters as independent variable.

**Parameter**	**Reference category or unit of measure**	***P*-value**	**Odds ratio**	**95% confidence interval**
Current smoking	No/Yes	<0.001	9.07	4.75, 17.3
Rural/urban region of habitation	Rural, 1; Urban, 2	<0.001	2.27	1.63, 3.16
History of menopause	No/Yes	0.03	1.86	1.07, 3.23
History of arthritis	No/Yes	<0.001	2.09	1.51, 2.89
History of cardiovascular diseases including stroke	No/Yes	0.001	1.71	1.23, 2.37
Depression score	Number	0.002	1.07	1.03, 1.11
Serum concentration of creatinine	mmol/L	0.04	0.99	0.98, 1.00

Asthma was present in 142 individuals indicating a prevalence of 2.6% (95%CI: 2.0, 3.1) in the study population (Table 6). For 132 individuals who could remember when their asthma was diagnosed, known duration of asthma was 17.2 ± 15.0 years (median: 14 years; range: 0–76 years). For 10 individuals, the known duration of asthma could not be well-remembered.

**Table 6:** Spirometric measurements and clinical signs of asthma (mean ± standard deviations; frequency; and 95% confidence intervals) in the Ural Eye ad Medical Study stratified by the presence of asthma.

	**Reference category or unit of measure**	**Asthma**	**No asthma**	*P*-value^*^
*n*		142	5,250	
Age	Years	62.8 ± 10.5	58.5 ± 10.5	<0.001
Forced vital capacity (FVC)	mL	1,905 ± 591	2,230 ± 538	<0.001
Forced expiratory volume in 1 s (FEV1)	mL	1,546 ± 552	1,910 ± 471	<0.001
FEV1/FVC	Ratio	0.80 ± 0.10	0.86 ± 0.06	<0.001
FEV1/FVC <0.7	Yes/Total	41/142	328/5,250	<0.001
Do you have chronic obstructive pulmonary disease?	Yes/No	52/142	283/5,250	<0.001
*n*		65	3,136	
How many times a day do you cough	Categories of: Never/1-3 times/4 – 6 times/>6 times	42%/37%/11%/11%	69%/26%/3%/2%	<0.001
How often have you have sputum during cough? (%)	Categories of: Never/1-3 times/4−6 times/>6 times	55%/31%/9%/5%	81%/16%/1%/1%	<0.001
How often have you been without sputum cough? (%)	Categories of: Never/1–3 times/4−6 times/>6 times	79%/15%/3%/3%	88%/11%/1%/1%	0.053
How often you do catch a cold in the winter? (%)	Categories of: Never/1–3 times/4−6 times/>6 times	28%/62%/11%	42%/55%/2%/0%	0.004
Is difficult for you to breathe when climbing stairs? (No/Yes)	Yes/No	19%/82%	52%/48%	<0.001
After how many steps do you have difficulty in breathing?	20–30/31–40/>40	58%/35%/8%	32%/42%/26%	<0.001
Do you have a fireplace or stove with an open fire at home? (No/Yes)	Yes/No	97%/3%	96%/4%	1.00
Is there smoke at your workplace? (No/Yes)	Yes/No	94%/6%	96%/4%	0.35
Is there dust at your workplace? (No/Yes)	Yes/No	92%/8%	94%/6%	0.60

* *Student-t-test, Chi-square test or analysis of variance ANOVA*.

Within the Russian ethnic group (*n* = 1,185), asthma was present in 27 individuals [2.5% (95%CI: 2.0, 3.0)] with a known duration of asthma of 14.9 ± 13.4 years (median: 11 years; range: 1–58 years). The Russian group and the non-Russian group within the study population did not differ significantly (*P* = 0.92) in the prevalence of asthma.

In univariate analysis, the prevalence of asthma was associated with older age (*P* < 0.001), female gender (*P* = 0.02), urban region of habitation (*P* = 0.01), smaller body height (*P* = 0.006), higher body mass index (*P* = 0.05), longer waist circumference (*P* = 0.01), and longer hip (*P* = 0.07) circumference, lower level of education (*P* = 0.02), lower prevalence of ownership of a two-wheeler (*P* = 0.002), less time spent on physically moderate to intensive activities during work (*P* = 0.048), or leisure time (*P* = 0.03), higher prevalence of a history of arterial hypertension (*P* = 0.001), arthritis (*P* = 0.006), backache (*P* = 0.009), therapy of dyslipidemia (*P* = 0.08), cancer (*P* = 0.01), cardiovascular disease including stroke (*P* = 0.01), diabetes (*P* = 0.009), headache (*P* = 0.02), menopause (*P* = 0.02), neck pain (*P* = 0.01), osteoarthritis (*P* < 0.001), thoracic spine pain (*P* < 0.001), tumbling (*P* = 0.08), unconsciousness (*P* = 0.004), higher blood concentrations of high-density lipoproteins (*P* = 0.007), urea (*P* = 0.01) and prothrombin complex (*P* = 0.006) and a lower international normalized ratio (*P* = 0.006), higher leucocyte count (*P* = 0.07), and higher percentage of basophile granulocytes (*P* = 0.02) and monocytes (*P* = 0.03), higher systolic blood pressure (*P* = 0.06), higher degree of meat processing for food (*P* = 0.04), lower amount of alcohol consumption (*P* = 0.008), higher prevalence of permanent stopping of alcohol consumption (*P* = 0.003), total hearing loss score (*P* = 0.002), higher depression score (*P* < 0.001) and anxiety score (*P* = 0.01), and weaker hand grip strength (*P* = 0.001).

In multivariable binary regression analysis, a higher prevalence of asthma remained to be significantly associated with less consumption of alcohol (OR: 0.53; 95%CI: 0.33, 0.87; *P* = 0.01), higher depression score (OR: 1.08; 95%CI: 1.03, 1.12; *P* < 0.001), and urban region of habitation (OR: 0.68; 95%CI: 0.49, 0.95; *P* = 0.0.03). In women, higher prevalence of asthma was additionally correlated with a higher prevalence of a history of menopause (OR: 2.03; 95%CI: 1.01, 4.08; *P* = 0.048).

## Discussion

The figure of the prevalence of airflow obstruction of 6.8% (using the definition of FEV1/FVC <0.70) in our Russian study population aged 40+years is the same as the figure of COPD found in the study by Andreeva et al. who examined 2,974 adults aged 35–70 years and residing North-western Russia (5). Andreeva et al. reported on a prevalence of COPD of 6.8% (95%CI: 5.8–7.9) as defined by the value of FEV1/FVC <0.70, and of 4.8% (95%CI: 3.9–5.7) as defined by the LLN-based cut-off. The prevalence of airflow obstruction using the LLN-based definition in our study population was 5.8% (95%CI: 5.2, 6.5). The airflow obstruction prevalence of 6.8% (using the definition of FEV1/FVC <0.70) in our Russian study population aged 40+years agrees also with the findings obtained previously in other study populations from other countries (19–29). Caballero et al. used a COPD definition based on spirometry and clinical symptoms and reported for Colombia a COPD prevalence of 8.9%, increasing with an age ≥60 years, male gender, history of tuberculosis, smoking, wood smoke exposure ≥10 years and very low education level (20). Zhong et al. used a post-bronchodilator FEV1/FVC ratio of <0.70 for the definition of COPD and found a COPD prevalence of 8.2% (men: 12.4%; women: 5.1%) in 20,245 Chinese aged 40+ years (29). The COPD prevalence was associated with rural region of habitation, older age, smoking, lower body mass index, lower level of education, and poor ventilation in the kitchen. van Gemert et al. performed a prospective observational cross-sectional study in rural Uganda and defining COPD as FEV1/FVC ratio of less than the lower limit of normal, found a prevalence of COPD of 16.2% (men: 15.4%, women: 16.8%) in a population of 588 individuals with a mean age of 45 ± 13.7 years, with 546 (93%) of the individuals being exposed to biomass smoke (27). The prevalence was highest in people aged 30–39 years, with the major risk factors of biomass smoke for both sexes and tobacco smoke for men. Jaganath et al. examined a population-based sample of 2,957 adults aged ≥35 years from four resource-poor settings in Peru (22). Defining COPD as a post-bronchodilator FEV1/FVC <0.70, they found an overall prevalence of COPD of 6.0% (95%CI: 5.1%, 6.8%) with the major risk factors of post-treatment tuberculosis and daily exposure to biomass fuel smoke. The prevalence of COPD (defined as post-bronchodilator FEV1/FVC ratio <0.7 and FEV1 <80% predicted) in Australians aged 40+ years was reported to be 7.5% among people with an age of 40+ years, and 29.2% among individuals with an age of 75+ years (25). Among individuals with an age of 40+ years, the prevalence of wheeze in the past 12 months was 30.0%, and the frequency of shortness of breath when hurrying on the level or climbing a slight hill was 25.2%. Other studies found a COPD prevalence of 16.2% in Uppsala/Sweden with the main risk factors of older age (OR: 2.08 per 10 years) and smoking (OR: 1.33 per 10 pack years), while higher education was protective (OR: 0.70 per 5 years of education) (21). The National Health and Nutrition Examination Survey (NHANES) in the U.S.A. reported on a COPD prevalence (defined as FEV1/FVC <0.70 or a FEV1/FVC smaller than the LLN) of 20.9% among individuals aged 40–79 years (24). Applying the same criterion to post-bronchodilator test results, prevalence was 14.0%. Using the LLN criterion and pre-bronchodilator spirometric test results, the COPD prevalence was 15.4%, while applying the same criterion to post-bronchodilator test results, the prevalence was 10.2%. In the study by Shahab et al. on 8215 adults aged 35+ years and participating in the Health Survey for England, prevalence of COPD as defined by spirometry was 13.3% (95%CI: 12.6, 14.0) with an unawareness rate of 80% (23). Even among individuals with severe or very severe COPD, only 46.8% reported any diagnosed respiratory disease. Smoking was strongly associated with COPD. In a study in Lisbon/Portugal on 710 participants, the overall weighted prevalence of GOLD stage II+ was 7.3% (95% C.I. 4.7, 11.3) (19). In a large nation-wide study performed in China on 50,991 adults aged 20 years or older, the prevalence of an airflow obstruction was 8.6% (95%CI: 7.5–9.9). The most comprehensive meta-analysis of the prevalence of COPD and its burden was recently performed by the Global Burden of Diseases Study which revealed a global age-standardized prevalence of 3.2% [95% uncertainty intervals (UI): 2·9–3·5] in men and 2.0% (95%UI: 1·8–2·1) in women (4). It roughly corresponds to a prevalence of a prevalence 6.8% in individuals aged 40+years as in our study population.

In the meta-analysis by the Global Burden of Diseases Study, smoking and ambient particulate matter were the main risks for COPD followed by household air pollution, occupational particulates, ozone, and second-hand smoke. These findings were congruent to those obtained in other investigations as well as in our study in which current smoking was by far the most important risk factor for airflow obstruction (OR: 9.07) (Table 1). In our study population, household air pollution and occupational particulates (questions: “Do you have a fireplace or stove with an open fire at home?”; “Is there smoke at your working place”; “Is there dust at your working place”) generally had a low frequency so that these parameters did not play a major role as risk factors for airflow obstruction in our study population, neither in the urban region or the rural area (Supplemental Table 1). In the investigation by Andreeva and associates, a higher prevalence of COPD was associated only with smoking (OR: 2.47; 95%CI: 1.60–3.82) (5).

The prevalence of asthma as found in our study population with a figure of 2.6% (95%CI: 2.0, 3.1) fits with previous in the literature (4). De Roos et al. found in the Rotterdam Study a prevalence of physician-diagnosed asthma of 3.6% (95% CI: 3.3%, 3.9%) in 14,621 participants with a mean age of 65.5 years, with a higher prevalence in women than in men (4.2 vs. 2.8%) (30). In a meta-analysis about the asthma prevalence in Iran, Varmaghani et al. reported an overall asthma prevalence of 4.56% (95% CI: 3.76%, 5.36%) among men, and of 4.17% (95% CI: 3.42–4.91%) among women (31). Factors associated with a higher asthma prevalence in our study population were a higher depression score and urban region of habitation. In the Global Burden of Disease Study, smoking and occupational asthmagens as the only quantified risk factors for asthma accounted for 16.5% of DALYs due to asthma (4).

Interestingly, the prevalence of airflow obstruction in men decreased from 17.2% (95% CI: 12.1, 22.4) in the age group of 40–44 years to 5.10% (95% CI: 3.10, 7.09) in the age group of 55–59 years, before it increased again (Table 3). A similar U-shaped form the association between airflow obstruction prevalence and age was not seen in the female study population (Table 3). The reasons for the lower airflow obstruction prevalence in middle-aged men have remained unclear so far. Potential factors might be an increased mortality of these individuals or measures taken to reduce the burden of airflow obstruction in elderly men.

Limitations of our study should be mentioned. First, the main limitation is that the definition we used for airflow obstruction was based only on the ratio FEV1/FCV <0.7 without that a bronchodilator test was performed. It is in contrast to the recommendation described in the GOLD Executive Summary 2017 and 2019 in which “a post-bronchodilator fixed ratio of FEV_1_/FVC <0.70 is the spirometric criterion for airflow limitation” (32, 33). In a similar manner, the GOLD Executive Summary 2013 stated that “although post-bronchodilator spirometry is required for the diagnosis and assessment of severity of COPD, the degree of reversibility of airflow limitation (e.g., measuring FEV_1_ before and after bronchodilator or corticosteroids) is no longer recommended.” (10). In the framework of a population-based study as ours it was however technically and logistically difficult to include the application of a bronchodilator into the routine procedures of a study on more than 5,000 participants. Second, since we did not apply bronchodilators we could not use the definition of GINA (Global Initiative for Asthma) for the definition of asthma (34). The difference in the methodology and definition of asthma might have influenced the results of the prevalence of asthma in our study population. Third, a point of concern is that the definition of COPD varies between studies (35). In our investigation we applied the definition of the Global Initiative of Chronic Obstructive Lung Disease with a ratio of FEV1 to FVC of <0.70, used however pre-bronchodilator values. Other studies used the lower limit of normal (LLN) method of deriving a threshold as the fifth percentile of the FEV1/FVC ratio in a healthy reference population. In our study, we additionally applied the LLN-based definition of airflow obstruction and, as compared to using the definition of FEV1/FVC <0.70, we arrived at similar results with respect to the associations between the prevalence of airflow obstruction and other parameters, while the prevalence of airflow obstruction defined by the LLN method was slightly lower than the prevalence of airflow obstruction defined by a value of FEV1/FVC <0.70 [5.8% (95%CI: 5.2, 6.5) vs. 6.8% (95%CI: 6.2, 7.5)]. One may also take into account that, both the GOLD definition and the LLN method may lead to misclassifications. In a study performed by Güder et al. the diagnostic accuracy and prognostic capability of the GOLD and LLN definition were compared to an expert-based diagnosis (36). It revealed that compared to the expert panel diagnosis, the GOLD-based definition of COPD led to a misclassification rate of 28%, and the three LLN-based definitions of COPD were associated with a misclassification rate of 46, 39, and 98%, respectively. In general, the GOLD-based definition was correlated with more false positive results, while the LLN-based definitions were associated with more false negative decisions. Fourth, most surveys of asthma used a case definition based on self-report of a diagnosis of asthma by a physician and wheeze (with other respiratory symptoms) in the past 12 months (37). In our study, asthma was defined by self-reported diagnosis of physician-made diagnosis of asthma. Toelle et al. and others have suggested that a case definition for clinically relevant asthma should preferably include wheezing symptoms occurring the preceding year and a bronchial hyper-responsiveness to inhalation of methacholine or histamine that is reversible with a bronchodilator (38). Several surveys have applied this definition to measure the asthma prevalence, but it has not been universally adopted. Reasons were logistical factors and the concern about a poor specificity and poor prediction of the future risk of asthma in individuals without symptoms (39). Pattemore et al. and Pekkanen and Pearce pointed out that the application of biological measurements to improve the validity of the asthma definition is associated with the goal of the investigation. To cite an example, the bronchial hyper-responsiveness has a similar or better specificity, but a worse sensitivity, than symptom questionnaires, so that it may be less suitable for the measurement of the prevalence (40, 41). Also, an overlap between COPD and asthma may have to be considered as assessed in the CHAIN study (42). Fifth, some subgroups of the study population were relatively small, such as the age subgroups with an age of 75–79 years or with an age of 80+years and the subgroup of individuals with asthma, so that the statistical power might not have been sufficient to detect the significance of associations for these subgroups (43).

In conclusion, in this typically ethically mixed urban and rural Russian population aged 40+ years, airflow obstruction prevalence was 6.8% (95%CI: 6.2, 7.5) with an awareness rate of 63.4% (95%CI: 58.5, 68.4) and the main risk factor of smoking. Household air pollution and occupational particulates did not play a major role as risk factors. Asthma prevalence was 2.6% (95%CI: 2.0, 3.1).

## Data Availability Statement

The datasets generated for this study are available on request to the corresponding author.

## Ethics Statement

The studies involving human participants were reviewed and approved by According to the Declaration of Helsinki, the Ethics Committee of the Academic Council of the Ufa Eye Research Institute approved the study and all participants gave informed written consent. The ethics committee confirmed that all methods were performed in accordance with the relevant guidelines and regulations. The patients/participants provided their written informed consent to participate in this study.

## Author Contributions

MB, GK, and JJ: design and conception. MB, GK, RZ, VS, TG, DY, YU, IA, SP-J, SM, RK, SA, IN, AZ, JJ, and NN: data assessment and editing and final approval of the manuscript. JJ: statistical analysis and writing of the manuscript.

### Conflict of Interest

The authors declare that the research was conducted in the absence of any commercial or financial relationships that could be construed as a potential conflict of interest.
