# Supplementary material for: Prevalence, Awareness, and Associated Factors of Airflow Obstruction in Russia: The Ural Eye and Medical Study
Source: Front Public Health. 2019 Nov 20;7:350. doi: 10.3389/fpubh.2019.00350 (PMC6879424; doi:10.3389/fpubh.2019.00350)
Supplement: Supplementary file 1 [file Data_Sheet_1.docx]

Supplemental Table 1

Demographic, socioeconomic, lifestyle-associated, and other parameters (mean ± standard deviations; frequency and 95% confidence intervals) in the Ural Eye ad Medical Study stratified by the presence of airflow obstruction (defined by a ratio of forced expiratory volume in one second divided by the mean forced vital capacity of <0.7) (Bikbov et al. 2019c).

| Parameter | | Reference Category or Unit of Measure | Forced expiratory volume in one second (FEV1) / Mean forced vital capacity (FVC) <0.7 | | FEV1/FVC ≥0.7 | *P*-Value* |
| --- | --- | --- | --- | --- | --- | --- |
| n | |  | 369 | | 5023 |  |
| Age (years) | | Years | 58.9 ± 11.0 | | 58.6 ± 10.5 | 0.55 |
| Gender | | Men / Women | 186 (50.4%) / 183 (49.6%) | | 2263 (45.1%) / 2760 (54.9%) | 0.051 |
| Region of habitation | | Rural / Urban | 198 (53.7%) / 171 (46.3%) | | 3198 (63.7%) / 1825 (36.3%) | <0.001 |
| Family status | | Married / Unmarried / Divorced / Widowed / Missing | 257 (69.6%) / 18 ( 4.9%) / 37 (10.0%) / 57 ( 15.4%) | | 3737 (74.4%) / 216 (4.3%) / 264 ( 5.3%) / 806 (16.0%) | 0.18 |
| Family status | | Married versus any other status | 257 (69.6%) / 112 (30.4%) | | 3737 (74.4%) / 1286 (25.6%) | 0.049 |
| Family type | | Joint (three generations) / nuclear (two generations) / single / family of 2 people | 100 (27.1%)/ 166 (45.0%) / 25 (6.8%) / 75 (20.3%) | | 1338 (26.6%) / 1943 (38.7%) / 298 (5.9%) / 1429 (28.4%) | 0.006 |
| Religion | | Muslim / Christian / Other | 205 (55.6%) / 160 (43.4%) | | 3251 (64.7%) / 1697 (33.8%) / 75 (1.5%) | 0.002 |
| Religion | | Muslim / any other religion | 205 (55.6%) / 164 (44.4%) | | 3251 (64.7%) / 1772 (35.3%) | <0.001 |
| Ethnicity | | Russian / Bashkirs / Tatars / Chuvash / Mari / Others / Missing | 101 (27.42%) / 67 (18.2) / 144 (39.0%) / 48 (13.0%) / 9 (2.4%) | | 1084 (21.6%) / 994 (19.8%) / 2291 (45.6%) / 538 (10.7%) / 21 (0.4%) / 95 (1.95) | 0.92 |
| Ethnicity | | Russian / any other ethnicity | 101 (27.42%) / 268 (72.6%) | | 1084 (21.6%) / 3939 (78.4%) | 0.01 |
| Body height | | Cm | 165.6 ± 8.8 | | 164.9 ± 8.8 | 0.16 |
| Body weight | | kg | 75.2 ± 14.6 | | 75.9 ± 14.6 | 0.35 |
| Body mass index | | kg/m^2^ | 27.4 ±5.0 | | 27.9 ± 5.0 | 0.07 |
| Waist circumference | | cm | 94.1 ± 13.0 | | 93.8 ± 13.3 | 0.63 |
| Hip circumference | | cm | 103.5 ± 11.9 | | 103.2 ± 12.7 | 0.60 |
| Socioeconomic parameters | | | | | | |
| Level of education | |  | 5.7 ± 1.6 | | 5.9 ± 1.8 | 0.046 |
| Monthly Income | | Below poverty line / average / above average / high | 97 (26.3%) / 264 (71.5%) / 8 (2.2%) / 0 | | 1189 (23.7%) / 3646 (72.6%) / 179 (3.6%) / 7 (0.1%) | 0.10 |
| Own ownership of house | | Yes / No | 365 (98.9%) / 4 (1.1%) | | 4968 (98.8%) / 59 (1.2%) | 1.00 |
| Own ownership of refrigerator | | Yes / No | 205 (99.5%) / 1 (0.5%) | | 1970 (99.2%) / 15 (0.8%) | 1.00 |
| Own ownership of second house | | Yes / No | 25 (15.3%) / 138 (84.7%) | | 556 (18.3%) / 2482 (81.7%) | 0.40 |
| Own ownership of telephone | | Yes / No | 341 (92.4%) / 28 (7.6%) | | 4341 (86.4%) / 682 (13.6%) | 0.001 |
| Own ownership of smartphone | | Yes / No | 79 (48.5%) / 84 (51.5%) | | 1669 (54.9%) / 1369 (45.1%) | 0.11 |
| Own ownership of television set | | Yes / No | 367 (99.5%) / 2 (0.5%) | | 4981 (99.2%) / 42 (0.8%) | 0.47 |
| Own ownership of car | | Yes / No | 98 (60.1%) / 65 (39.9%) | | 2120 (69.8%) / 918 (30.2%) | 0.02 |
| Own ownership of two-wheeler | | Yes / No | 115 (31.2) / 254 (68.8%) | | 2137 (42.6%) / 2885 (57.4%) | <0.001 |
| Own ownership of tractor | | Yes / No | 7 (3.4%) / 199 (96.6%) | | 69 (4.3%) / 1899 (95.7%) | 0.51 |
| Own ownership of bullock cart | | Yes / No | 10 (4.9%) / 196 (95.1%) | | 69 (3.5%) / 1915 (96.5%) | 0.38 |
| Own ownership of computer | | Yes / No | 96 (58.9%) / 67 (41.1%) | | 2051 (67.5%) / 987 (32.5%) | 0.03 |
| Physical activity | | | | | | |
| How long is your usual work day? | | Minutes | 483 ± 262 | | 457 ± 262 | 0.14 |
| Does your work involve mostly sitting or standing with less than 10 minutes of walking at a time? (Yes / No) | | Yes / No | 211 (65.7%) / 110 (34.3%) | | 3110 (70.8%) / 1280 (29.2%) | 0.06 |
| Does your work involve physically vigorous activity (like heavy lifting or digging) or physically moderate intensity activity (like brisk walking or carrying light loads) (Yes / No) | | Yes / No | 190 (59.4%) / 130 (40.6%) | | 2539 (57.9%) / 1847 (42.1%) | 0.64 |
| How many days a week do you do such physically vigorous activity during work? (Yes / No) | | Yes / No | 5.7 ± 1.5 | | 5.7 ± 1.5 | 0.93 |
| On a usual day how much time do you spend on such physically vigorous work during work? | | Minutes | 225 ± 291 | | 217 ± 273 | 0.62 |
| Does your work involve physically moderate-intensive activity, like brisk walking or carrying light loads for at least 10 minutes at a time? | | Yes / No | 216 (68.6%) / 99 (31.4%) | | 3024 (69.0%) / 1361 (31.0) | 0.90 |
| In a typical week, on how many days do you do physically moderate to intensive activities as part of your work? | | Number | 5.8 ± 1.6 | | 5.8 ± 1.5 | 0.92 |
| Per mean day including all days of the week, how much time do you spend with physically moderate to intensive activities as part of your work? | |  | 202 ± 254 | | 198 ± 243 | 0.75 |
| Do you walk or use a bicycle (pedal cycle) for at least 10 minutes continuously to get to and from places? | | Yes / No | 317 (88.3%) / 42 (11.7%) | | 4545 (91.7%) / 413 (8.3%) | 0.03 |
| In a typical week, on how many days do you walk or bicycle for at least 10 minutes to go to and from places? | |  | 6.3 ± 1.5 | | 6.4 ± 1.3 | 0.15 |
| How much time do you spend walking or bicycling for travel in a day? | |  | 162 ± 155 | | 153 ± 142 | 0.32 |
| Does your recreation, sport or leisure time involve mostly sitting, reclining or standing activities, with no physical activity lasting more than 10 minutes at a time? | | Yes / No | 241 (65.5%) / 127 (34.5%) | | 3155 (63.4%) / 1825 (36.6%) | 0.43 |
| In your leisure time, do you do any physically vigorous activities like running, strenuous sports or weight lifting for at least 10 minutes at a time? | | Yes / No | 81 (22.4%) / 281 (77.6%) | | 1459 (29.6%) / 3464 (70.4%) | 0.003 |
| If yes, In a typical week, on how many days do you do physically vigorous activities as part of your leisure time? | |  | 5.8 ± 2.0 | | 5.9 ± 1.9 | 0.56 |
| How much time do you spend on physically vigorous activities as part of your leisure time on a typical day? | |  | 185 ± 139 | | 174 ± 150 | 0.46 |
| In your leisure time, do you do any moderate intensity activities like brisk walking, cycling or swimming for at least 10 minutes at a time? | | Yes / No | 146 (41.5%) / 206 (58.5%) | | 2293 (46.9%) / 2600 (53.1%) | 0.053 |
| In a typical week, on how many days do you do physically moderate to intensive activities as part of your leisure time? | |  | 5.2 ± 2.3 | | 5.4 ± 2.2 | 0.42 |
| How much time do you spend on physically moderate to intensive activities per day of week during your leisure time? (Minutes) | |  | 59.5 ± 107.0 | | 72.8 ± 117.5 | 0.02 |
| Over the past 7 days, how much time did you spend sitting or reclining on a typical day? | |  | 1096 ± 929 | | 1055 ± 946 | 0.42 |
| History of diseases | | | | | | |
| History of arterial hypertension | | Yes / No | 166 (45.0) / 203 (55.0%) | | 1963 (39.1%) / 3060 (60.9%) | 0.03 |
| History of arthritis | | Yes / No | 138 (37.4%) / 231 (62.6%) | | 1265 (25.2%) / 3758 (74.8%) | <0.001 |
| History of backache | | Yes / No | 237 (64.2%) / 132 (35.8%) | | 2671 (53.2%) / 2352 (46.8%) | <0.001 |
| History of therapy of hyperlipidemia | | Yes / No | 49 (14.7%) / 285 (85.3%) | | 386 (82.%) / 4337 (91.8%) | <0.001 |
| History of cancer | | Yes / No | 12 (3.3%) / 357 (96.7%) | | 140 (2.8%) / 4883 (97.2%) | 0.62 |
| History of cardiovascular disorders including stroke | | Yes / No | 145 (39.3%) / 224 (60.7%) | | 1321 (26.3%) / 3702 (73.7%) | <0.001 |
| History of dementia | | Yes / No | 4 (12.2%) / 324 (87.8%) | | 33 (0.7%) / 4990 (99.3%) | 0.32 |
| History of diabetes mellitus | | Yes / No | 45 (12.2%) / 324 887.8%) | | 396 (7.9%) / 4626 (92.1%) | 0.006 |
| History of diarrhea | | Yes / No | 3 (0.8%) / 366 (99.2%) | | 24 (0.5%) / 4999 | 0.43 |
| History of bone fracture | | Yes / No | 151 (40.9%) / 218 (59.1%) | | 1497 (29.8%) / 3526 (70.2%) | <0.001 |
| History of headache | | Yes / No | 194 (52.6%) / 175 (47.4%) | | 2350 (46.8%) / 2673 (53.2%) | 0.04 |
| History of heart attack | | Yes / No | 27 (7.3%) / 342 (92.7%) | | 259 (5.2%) / 4764 (94.8%) | 0.09 |
| History of iron-deficiency anemia | | Yes / No | 34 (9.2%) / 335 (90.8%) | | 270 (5.4%) / 4753 (94.6%) | 0.005 |
| History of low blood pressure and hospital admittance | | Yes / No | 22 (6.0%) / 347 (94.0%) | | 157 (3.1%) / 4846 (96.9%) | 0.006 |
| History of neck pain | | Yes / No | 135 (36.6%) / 234 (63.4%) | | 1434 (28.5%) / 3589 (71.5%) | 0.001 |
| History of osteoarthritis | | Yes / No | 102 (27.6%) / 267 (72.4%) | | 887 (17.7%) / 4136 (82.3%) | <0.001 |
| History of thoracic spine pain | | Yes / No | 115 (31.2%) / 254 (68.8%) | | 1155 (23.0%) / 3868 (77.0%) | 0.001 |
| History of skin disease | | Yes / No | 30 (8.1%) / 339 (91.9%) | | 256 (5.1%) / 4767 (94.9%) | 0.02 |
| History of use of steroids | | Yes / No | 0 / 369 (100%) | | 20 (0.4%) / 5001 (99.6%) | 0.40 |
| History of thyreopathy | | Yes / No | 54 (14.6%) / 315 (85.4%) | | 468 (9.3%) / 4555 (90.7%) | 0.002 |
| History of tumbling | | Yes / No | 112 (30.4%) / 257 (69.6%) | | 946 (18.8%) / 4077 (81.2%) | <0.001 |
| History of unconsciousness | | Yes / No | 47 (12.7%) / 332 (87.3%) | | 396 (7.9%) / 4627 (92.1%) | 0.002 |
| Age of the last menstrual bleeding (years) | | Years | 48.6 ± 4.7 | | 48.3 ± 5.0 | 0.39 |
| Age of last regular menstrual bleeding (years) | | Years | 48.4 ± 4.7 | | 48.1 ± 5.0 | 0.45 |
| Menopause | |  | 164 (90.6%) / 17 (9.4%) | | 2184 (80.0%) / 546 (20.0%) | <0.001 |
| Blood concentrations (mmol/L) of: | | | | | | |
| Alanine aminotransferase | | IU/L | 21.5 ± 12.0 | | 21.2 ± 12.5 | 0.67 |
| Aspartate aminotransferase | | IU/L | 20.8 ± 9.5 | | 20.9 ± 11.4 | 0.86 |
| Bilirubin, total | | µmol/L | 13.4 ± 11.8 | | 15.4 ± 11.5 | 0.002 |
| High-density lipoproteins | | mmol/L | 2.36 ± 0.99 | | 2.31 ± 0.89 | 0.36 |
| Low-density lipoproteins | | mmol/L | 2.01 ± 1.40 | | 2.14 ± 1.18 | 0.10 |
| Triglycerides | | mmol/L | 1.39 ± 0.63 | | 1.41 ± 0.76 | 0.45 |
| Cholesterol | | mmol/L | 5.75 ± 1.97 | | 5.84 ± 1.67 | 0.39 |
| C-reactive protein | | mg/L | 7.45 ± 2.46 | | 7.01 ± 2.32 | 0.59 |
| Rheumatoid factor | | IU/Ml |  | |  |  |
| Erythrocyte sedimentation rate | | mm / hour | 13.5 ± 11.6 | | 14.0 ± 11.2 | 0.45 |
| Glucose | | mmol/L | 4.85 ± 1.90 | | 5.00 ± 1.64 | 0.16 |
| Creatinine | | µmol/L | 85.4 ± 21.4 | | 91.3 ± 25.5 | <0.001 |
| Urea | | mmol/L | 5.15 ± 1.38 | | 5.05 ± 1.49 | 0.18 |
| Residual nitrogen | | g/L | 0.25 ± 0.05 | | 0.25 ± 0.08 | 0.33 |
| Total protein | | g/L | 75.6 ± 6.0 | | 76.0 ± 6.5 | 0.27 |
| Color index | | Units |  | |  |  |
| International normalized ratio | | INR | 1.05 ± 0.12 | | 1.06 ± 0.15 | 0.004 |
| Blood clotting time | | Minutes |  | |  |  |
| Prothrombin index | | % |  | |  |  |
| Hemoglobin | | g/Dl | 144.5 ± 15.7 | | 142.7 ± 14.7 | 0.03 |
| Erythrocytes | | 10^6^ cells / µL | 4.54 ± 0.39 | | 4.49 ± 0.38 | 0.04 |
| Leukocytes | | 10^9^ cells / L | 5.25 ± 1.66 | | 5.09 ± 1.40 | 0.08 |
| Rod-core granulocyte | | % of Leukocytes | 2.38 ± 1.53 | | 2.43 ± 1.50 | 0.56 |
| Segment nuclear granulocyte | | % of Leukocytes | 59.7 ± 7.8 | | 59.4 ± 7.3 | 0.46 |
| Eosinophil granulocytes | | % of Leukocytes | 2.08 ± 1.00 | | 2.09 ± 1.18 | 0.89 |
| Basophile granulocytes | | % of Leukocytes | 1.02 ± 0.15 | | 1.02 ± 0.15 | 0.73 |
| Lymphocytes | | % of Leukocytes | 31.8 ± 8.4 | | 31.9 ± 6.3 | 0.87 |
| Monocytes | | % of Leukocytes | 5.28 ± 3.01 | | 5.20 ± 2.23 | 0.62 |
| Blood pressure, systolic | | mmHg | 131.6 ± 19.5 | | 133.6 ± 20.1 | 0.21 |
| Blood pressure, diastolic | | mmHg | 81.9 ± 82.2 | | 82.1 ± 10.3 | 0.63 |
| Blood pressure, mean | | mmHg | 98.3 ± 12.6 | | 99.7 ± 12.4 | 0.18 |
| Ankle-brachial index, right side | | Ratio | 1.29 ± 0.19 | | 1.27 ± 0.19 | 0.20 |
| Ankle-brachial index, left side | | Ratio | 1.28 ± 0.19 | | 1.26 ±0.19 | 0.17 |
| Medical Doctor seen within the last year | | Yes / No | 273 (74.0%) / 96 (26.0%) | | 3851 (76.7%) / 1172 (23.3%) | 0.25 |
| Diet | | | | | | |
| Diet | | Vegetarian / Mixed | 0 / 369 (100%) | | 7 (0.1%) / 5017 (99.9%) | 1.00 |
| Number of meals per day | | Number | 3.55 ± 0.79 | | 3.63 ± 0.79 | 0.04 |
| In a week how many days do you eat fruits? | | Number | 5.0 ± 2.1 | | 5.4 ± 2.0 | 0.002 |
| How many servings of fruit do you take on one of those days? | | g | 189 ± 116 | | 185 ± 97 | 0.51 |
| In a week how many days do you eat vegetables? | | Number | 6.0 ± 1.6 | | 6.3 ± 1.4 | 0.001 |
| How many servings of vegetables do you eat on one of those days? | | g | 250 ± 110 | | 244 ± 113 | 0.30 |
| Type of oil used for cooking | | Vegetable oil / Non-vegetable Oil | 268 (95.7%) / 10 (3.6%) | | 4192 (95.9%) / 141 (3.2%) | 0.54 |
| Food containing whole grains | | Yes / No | 273 (74.0%) / 96 (26.0%) | | 4016 (80.0%)/ 1004 (20.0) | 0.007 |
| Salt consumed per day | | G | 4.1 ± 2.5 | | 4.3 ± 2.4 | 0.26 |
| Degree of processing of meat | | Weak / Medium / Well Done | 13 (3.5%) / 144 (39.0% / 212 (57.5%) | | 106 (2.1%) / 1772 (35.3%) / 3140 (62.6%) | 0.03 |
| Smoking | | | | | | |
| Do you currently smoke any tobacco products? | | Yes / No | 150 (40.7%) / 219 (59.3%) | | 550 (11.0%) / 4470 (89.0%) | <0.001 |
| Do you smoke daily? | | Yes / No | 148 (40.1%) / 221 (59.9%) | | 529 (10.5%) / 4494 (89.5%) | <0.001 |
| How old were you when you first started smoking? | | Years | 19.6 ± 7.5 | | 19.1 ± 5.9 | 0.45 |
| Have you stopped smoking? (yes / no) | | Yes / No | 28 (5.0%) / 530 (95.0%) | | 6 (4.0%) / 144 (96.0%) | 0.83 |
| How many cigarettes do smoke each day? | | 0 / ≤10 / 11-20 / 21-30 / >30 | 216 (58.7%) / 32 (8.7%) / 49 (13.3%) / 50 (13.6%) /21 (5.7%) | | 4463 (89.2%) / 151 (3.0%) / 352 (7.0%) / 30 (0.6%) / 10 (0.2%) | <0.001 |
| Package years (package = 20 cigarettes) | | Number | 16.8 ± 26.2 | | 3.4 ± 11.0 | <0.001 |
| How much time after awakening do you smoke the first cigarette of the day? | | <5 minutes / 6-30 min. / 31 – 60 min. / >60 min | 58 (38.7%) / 56 (37.3%) / 22 14.7%) /14 (9.3%) | | 131 (24.3%) / 228 (42.3%) / 95 (17.6%) / 85 (15.8%) | 0.001 |
| Difficult to refrain from smoking in forbidden places? | | Yes / No | 29 (19.1%) / 123 (80.9%) | | 70 (12.7%) / 482 (87.3%) | 0.03 |
| Which cigarette would you hate to give up? (First one in the morning) | | Yes / No | 25 (16.7%) / 125 883.3%) | | 84 (15.5%) / 459 (84.5%) | 0.71 |
| Do you smoke more frequently during the first hours after waking than during the rest of the day? | | Yes / No | 46 (30.3%) / 106 (69.7%) | | 125 (22.7%) / 426 (77.3%) | 0.07 |
| Do you smoke when you ill? | | Yes / No | 53 (34.9%) / 99 (65.1%) | | 154 (28.0%) / 396 (72.0%) | 0.11 |
|  | Alcohol | | | | | |
| Alcohol consumed such as beer, whisky, rum, gin brandy or other local products? (yes / no) | | Yes / No | 71 (24.7%) / 278 (75.3%) | | 1130 (22.5%) / 3893 (77.5%) | 0.33 |
| Age when you first started to drink alcohol? | |  | 20.5 ± 5.2 | | 20.4 ± 5.1 | 0.83 |
| Did you stop drinking alcohol and are you still completely abstinent? | | Yes / No | 17 (16.3%) / 87 (83.7%) | | 289 (20.2%) / 1144 (79.8%) | 0.38 |
| Age when you stopped drinking alcohol? | | Years | 49.5 ± 9.8 | | 44.6 ± 12.8 | 0.12 |
| How many alcoholic drinks do you have on a typical day when you are drinking? | | Number | 225 ± 216 | | 193 ± 147 | 0.15 |
| How often do you have 6 or more drinks on one occasion? | | Never / Rarely / Sometimes / Often / Cannot Say | 40 (54.8%) / 19 (26.0%) / 13 (17.8%) / 1 (1.4%) | | 653 (62.3%) / 257 (24.5%) / 121 (11.5%) / 17 (1.6%) | 0.18 |
| How often during the last year have you found that you were not able to stop drinking once you had started? | | Never / Rarely / Sometimes / Often / Cannot Say | 51 (75.0%) / 10 (14.7%9 / 6 (8.8%) / 1 (1.5%) | | 866 (87.7%) / 106 (10.7%) / 12 (1.2%) / 3 (0.3%) | 0.01 |
| How often during the last year have you failed to do what was normally expected from you because of drinking? | | Never / Rarely / Sometimes / Often / Cannot Say | 54 (81.8%) / 11 (16.7%) / 1 (1.5%) | | 884 (89.2%) / 94 (9.5%) / 11 (1.1%) / 2 (0.2%) | 0.19 |
| How often during the last year have you needed a first drink in the morning to get yourself going after a heavy drinking? | | Never / Rarely / Sometimes / Often / Cannot Say | 54 (80.6%) / 12 (17.9%) / 1 (1.5%) | | 885 (87.8%) / 104 (10.3%) / 16 (1.6%) / 3 (0.3%) | 0.25 |
| How often during the last year have you had a feeling of guilt or remorse after drinking? | | Never / Rarely / Sometimes / Often / Cannot Say | 52 74.3%) / 12 (17.1%) / 6 (8.6%) | | 883 (87.3%) / 99 (9.8%) / 22 (2.2%) /8 (0.8%) | 0.02 |
| How often during the last year have you been unable to remember what happened the last night? | | Never / Rarely / Sometimes / Often / Cannot Say | 53 (79.1%) / 13 (19.4%) / 1 (1.5%) | | 897 (89.0%) / 93 (9.2%) / 16 (1.6%) / 2 (0.2%) | 0.10 |
| Have you or someone else has been injured as a result of your drinking? | | Yes / No | 2 (2.0%) / 96 (98.0%) | | 2 (0.2%) / 1181 (99.8%) | 0.03 |
| Has a relative, friend or a doctor or another health worker been concerned about your drinking or suggested you to drink less? | | Yes / No | 3 (3.1%) / 95 /96.9%) | | 15 (1.3%) / 1168 (98.7%) | 0.15 |
| Hearing loss | | | | | | |
| Do you experience the hearing loss | | No / Sometimes / Yes | 244 (66.1%) / 15 (4.1%9 / 110 (29.8%) | | 3528 (70.2%) / 203 (4.0%) / 1292 (25.7%) | 0.10 |
| Does a hearing problem cause you to feel embarrassed when meeting new people? | | No / Sometimes / Yes | 321 (87.0%) / 18 (4.9%) / 30 (8.1%) | | 4326 (86.1%) / 212 (4.2%)/ 484 (9.6%) | 0.45 |
| Does a hearing problem cause you to feel frustrated when talking to members of your family? | | No / Sometimes / Yes | 319 (86.4%) / 15 (4.1%) / 35 (9.5%) | | 4297 (85.5%) / 232 (4.6%) / 494 (9.8%) | 0.70 |
| Do you have difficulties in hearing when someone speaks in a whisper tone? | | No / Sometimes / Yes | 281 (76.2%) / 16 (4.3%) / 72 (19.5%) | | 4112 (81.9%) / 192 (3.8%) / 719 (14.3%) | 0.01 |
| Do you feel handicapped by a hearing problem? | | No / Sometimes / Yes | 327 (88.6%) / 13 (3.5%) / 29 (7.9%) | | 4488 (89.3%) / 187 (3.7%) / 348 (6.9%) | 0.58 |
| Does a hearing problem cause you difficulties when visiting friends, relatives, or neighbors? | | No / Sometimes / Yes | 324 (87.8%) / 21 (5.7%) / 24 (6.5%) | | 4440 (88.4%) / 224 (4.5%) / 359 (7.1%) | 0.99 |
| Does a hearing problem cause you to attend religious services less often than you would like? | | No / Sometimes / Yes | 344 (93.2%) / 11 (3.0%) / 14 (3.8%) | | 4612 (91.8%) / 154 (3.1%) / 257 (5.1%) | 0.23 |
| Does a hearing problem cause you to have arguments with family members? | | No / Sometimes / Yes | 336 (91.1%) / 12 (3.3%) / 21 (5.7%) | | 4488 (89.3%) / 203 (4.0%) / 332 (6.6%) | 0.32 |
| Does a hearing problem cause you to have difficulties when listening to TV or radio? | | No / Sometimes / Yes | 306 (82.9%) / 17 (4.6%) / 46 (12.5%) | | 4302 (85.6%) / 213 (4.2%) / 508 (10.1%) | 0.16 |
| Do you feel any difficulty with your hearing limits hampering your personal or social life? | | No / Sometimes / Yes | 331 (89.7%) / 16 (4.3%) / 22 (6.0%) | | 4536 (90.3%) / 183 (3.6%) / 304 (6.1%) | 0.85 |
| Does a hearing problem cause you difficulties when in a restaurant with relatives or friends? | | No / Sometimes / Yes | 342 (92.7%) / 12 (3.3%) / 15 (4.1%) | | 4582 (91.2%) / 170 (3.4%) / 271 (5.4%) | 0.23 |
| Hearing Loss Total Score | | Sum | 5.4 ± 10.5 | | 5.1 ± 11.0 | 0.55 |
| Webers test | | > Right Eye / > Left Eye / Equal | 10 (2.7%) / 13 (3.5%) / 346 893.8%) | | 107 (2.1%) / 79 (1.6%) / 4837 (96.3%) | 0.12 |
| Rinne test right ear, positive | | Yes / No | 180 (48.9%) / 188 (51.1%) | | 1890 (37.8%) / 3113 (62.2%) | <0.001 |
| Rinne test left ear, positive | | Yes / No | 177 (48.2%) / 190 (51.8%) | | 1866 (37.3%) / 3140 (62.7%) | <0.001 |
| Depression | | | | | | |
| I was bothered by things that usually don't bother me. | | Yes / No | | 185 (50.1%) / 184 (49.9%) | 2267 (45.1%) / 2755 (54.9%) | 0.07 |
| I did not feel like eating, my appetite was poor | | Yes / No | | 110 (29.8%) / 259 (70.2%) | 1417 (28.2%) / 3605 (71.8%) | 0.51 |
| I felt that I could not shake off the blues, even with the help from family and friends | | Yes / No | | 99 (26.8%) / 270 (73.2%) | 957 (19.1%) / 4065 (80.9%) | 0.001 |
| I felt that I was just as good as other people | | Yes / No | | 308 (83.5%) / 61 (16.5%) | 4257 (84.8%) / 765 (15.2%) | 0.50 |
| I had trouble keeping my mind on what I was doing | | Yes / No | | 142 (38.5%) / 227 (61.5%) | 1596 (31.8%) / 3426 (68.2%) | 0.009 |
| I felt depressed | | Yes / No | | 155 (42.0%) / 214 (58.0%) | 1637 (32.6%) / 3385 (67.4%) | <0.001 |
| I felt that everything I did was an effort | | Yes / No | | 146 (39.6%) / 223 (60.4%) | 1659 (33.0%) / 3363 (67.0%) | 0.01 |
| I felt hopeful about the future | | Yes / No | | 334 (90.5%) / 36 (9.5%) | 4658 (92.8%) / 364 (7.2%) | 0.12 |
| I thought my life had been a failure | | Yes / No | | 53 (14.4%) / 316 (85.6%) | 562 (11.2%) / 4460 (88.8%) | 0.07 |
| I felt fearful | | Yes / No | | 110 (29.8%) / 259 (70.2%) | 1358 (27.0%) / 3664 (73.0%) | 0.25 |
| My sleep was restless | | Yes / No | | 180 (48.8%) / 189 (51.2%) | 2298 (45.8%) / 2724 (54.2%) | 0.28 |
| I was happy | | Yes / No | | 337 (91.3%) / 32 (8.7%) | 4784 (95.3%) / 238 (4.7%) | 0.002 |
| I talked less than usual | | Yes / No | | 189 (51.2%) / 180 (48.8%) | 2146 (42.7%) / 2876 (57.3%) | 0.002 |
| I felt lonely | | Yes / No | | 89 (24.1%) / 280 (75.9%) | 844 (16.8%) / 4178 (83.2%) | 0.001 |
| People were unfriendly | | Yes / No | | 94 (25.7%) / 274 (74.3%) | 898 (17.9%) / 4124 (82.1%) | <0.001 |
| I enjoyed life | | Yes / No | | 336 (91.1%) / 33 (8.9%) | 4651 (92.6%) / 371 (7.4%) | 0.26 |
| I had crying spells | | Yes / No | | 112 (30.4%) / 257 (69.9%) | 1366 (27.2%) / 3656 72.8%) | 0.20 |
| I felt sad | | Yes / No | | 239 (64.8%) / 130 (35.2%) | 2908 (57.9%) / 2114 (42.1%) | 0.01 |
| I felt that people dislike me | | Yes / No | | 73 (19.8%) / 296 (80.2%) | 780 (15.5%) / 4242 (84.5%) | 0.04 |
| I could not get "going" | | Yes / No | | 95 (25.7%) / 274 /74.3%) | 1072 (21.3%) / 3950 (78.7%) | 0.05 |
| Depression score (adapted) | | Score | | 2.05 ± 4.1 | 1.08 ± 3.73 | <0.001 |
| State-Trait Anxiety Inventory (STAI) | | | | | | |
| I feel pleasant | | Yes / No | | 353 (95.7%) / 16 (4.3%) | 4782 (95.2%) / 240 (4.8%) | 0.80 |
| I tire quickly | | Yes / No | | 195 (52.8%) / 174 (47.2%) | 2331 (46.4%) / 2691 (53.6%) | 0.02 |
| I feel like crying | | Yes / No | | 87 (23.6%) / 282 (76.4%) | 1121 (22.3%) / 3901 (77.7%) | 0.56 |
| I wish I could be as happy as others seem to be | | Yes / No | | 239 (64.8%) / 130 (35.2%) | 3064 (61.0%) / 1957 (39.0%) | 0.17 |
| I am losing out on things because I can't make up my mind soon enough | | Yes / No | | 97 (26.3%) / 272 (73.7%) | 1079 (21.5%) / 3943 (78.5%) | 0.04 |
| I feel rested | | Yes / No | | 274 (74.3%) / 95 25.7%) | 3920 (78.1%) / 1102 (21.9%) | 0.09 |
| I am calm, cool and collected | | Yes / No | | 304 (82.4%) / 65 (17.6%) | 4403 (87.7%) / 619 (12.3%) | 0.004 |
| I feel that difficulties are piling up so that I can't overcome them | | Yes / No | | 107 (29.0%) / 262 (71.0%) | 1186 (23.6%) / 3836 (76.4%) | 0.02 |
| I worry too much over something that really doesn't matter | | Yes / No | | 143 (38.8%) / 226 (61.2%) | 1996 (39.7%) / 3026 (60.3%) | 0.74 |
| I am happy | | Yes / No | | 333 (90.2%) / 16 (9.2%) | 4763 (94.8%) / 259 (5.2%) | 0.001 |
| I am inclined to take things hard | | Yes / No | | 309 (83.7%) / 60 (16.3%) | 3807 (75.8%)/ 1215 (24.2%) | <0.001 |
| I lack self-confidence | | Yes / No | | 148 (40.1%) / 221 (59.9%) | 1934 (38.5%) / 3088 (61.5%) | 0.54 |
| I feel safe | | Yes / No | | 283 (76.7%) / 86 (23.3%) | 3896 (77.6%) / 1126 (22.4%) | 0.70 |
| I try to avoid facing a crises or difficulty | | Yes / No | | 294 (79.7%) / 75 (20.3%) | 3864 (76.9%) / 1158 (23.1%) | 0.25 |
| I feel blue | | Yes / No | | 120 (32.5%) / 249 (67.5%) | 1518 (30.2%) / 3504 (69.8%) | 0.35 |
| I am content | | Yes / No | | 333 (90.2%)/ 36 (9.8%) | 4654 (92,7%) / 368 (7.3%) | 0.10 |
| Some unimportant thoughts run through my mind and bother me | | Yes / No | | 212 (57.5%) / 157 (42.5%) | 2381 (47.4%) / 2641 (52.6%) | <0.001 |
| I take disappointments so keenly that I can't put them out of my mind | | Yes / No | | 136 (36.9%) / 233 (63.1%) | 1610 32.1%) / 3412 (67.9%) | 0.07 |
| I am a steady person | | Yes / No | | 347 (94.0%) / 22 (6.0%) | 4556 (90.7%) / 466 (9.3%) | 0.03 |
| I get in a state of tension or turmoil as I think over my recent concerns and interests | | Yes / No | | 133 (36.0%) / 236 (64.0%) | 1369 (27.3%) / 3653 (72.7%) | <0.001 |
| State-Trait Anxiety Inventory (STAI) Score (adapted) | | Yes / No | | -0.02 ± 3.80 | -0.74 ± 3.52 | <0.001 |
| I attempted suicide due to financial reasons then | | Yes / No | | 0% | 11 (0.2%) / 5011 (99.8%) | 1.00 |
| Have you thought of committing suicide in the last 6 months or earlier? | | Yes / No | | 1 (0.3%) / 368 (99.7%) | 24 (0.5%) / 4998 (99.5%) | 1.00 |
| I thоught of suicide | | Yes / No | | 3 (0.8%) / 366 (99.2%) | 61 (1.2%) / 4961 (98.8%) | 0.80 |
| I thоught of suicide due to financial reasons then | | Yes / No | | 2 (0.5%) / 367 (99.5%) | 10 (0.2%) / 5012 (99.8%) | 0.20 |
| Dynamometry | |  | | | | |
| Manual dynamometry, right hand | | dekaNewton | | 30.3 ± 11.8 | 30.6 ± 11.7 | 0.68 |
| Manual dynamometry, left hand | | dekaNewton | | 27.0 ±11.3 | 26.4 ± 11.6 | 0.35 |

* Student-t-test, Chi-square test or analysis of variance ANOVA
